# Research on the deformation laws of buildings adjacent to shield tunnels in clay strata

**DOI:** 10.1038/s41598-023-50855-1

**Published:** 2024-01-02

**Authors:** Liping Cai, Kang Shi, Feng Jiang, Guanyi Chen, Zhiyong Xiao, Chengcheng Zheng, Shuai Zhang, Yue Wu

**Affiliations:** 1 Shandong University of Science and Technology, Qingdao, 266590 China; 2 Qingdao City University, Qingdao, 266106 China; 3 Guangdong Honggao Construction Group, Dongguan, 523000 China; 4 Changsha University of Science and Technology, Changsha, 410114 China; 5 Ludong University, Yantai, 264025 China

**Keywords:** Civil engineering, Scientific data

## Abstract

Earth pressure shields are widely used in tunnel construction due to their low environmental impact and mechanized operations. However, ensuring the stability of the excavation surface during the construction process is crucial. Any instability in the excavation surface can lead to soil destruction, such as body collapse or surface uplift. Additionally, the tunneling process can cause deformation disturbances to nearby buildings. In the case of Beijing Metro Line 17, detailed survey data and construction monitoring data were collected through field surveys and tests. The study combined theoretical analysis and numerical simulations to investigate the impact of shield tunneling in clay layers on neighboring buildings. The focus was on analyzing the physical deformation and the response law of influencing factors, such as stratum parameters and engineering effects on surface settlement, building inclination, and distortion. Furthermore, sensitivity analysis of the deformation impact was conducted, and corresponding measures for deformation control were proposed.

## Introduction

At present, space resources on the ground are scarce, so in order to better relieve traffic pressure, we need to focus on the utilization of underground space. With the development of society and technological progress, metro technology has become the mainstream of transport development. There are many kinds of underground construction methods, compared with other tunnel excavation methods, shield tunnel causes relatively little damage to the ground, but because of geological conditions and some construction process is not standardised enough, its excavation often results in surface settlement and the deformation of surrounding buildings. For these phenomena, many scholars began to make further research on the construction-induced surface settlement and building deformation. Verruijt^1^ proposed the mirror-image method, assuming that the excavated soil has the same linear elastic material and physical properties, and uses the principle of elastic plane analysis to derive the vertical and horizontal displacements generated by shield construction. Sagaseta^2^ used the isotropy of each, incompressibility of the assumed tunnel perimeter rock to derive the equation for the three-dimensional settlement of the ground surface. Loganathan^3^ proposed two scenarios of perimeter rock loss, i.e., the loss that occurs immediately after excavation without considering drainage and the loss that occurs when consolidation and creep are considered. Sun^4^ derived a viscoelastic-plasticity theory for enclosed rock of lined and circular tunnels, utilizing a combination of viscoelastic theory and practical engineering. Yao^5^ studied the settlement law of building foundations by applying a vertical uniform load transformed by self-weight on the foundations of buildings. Zhang^6^ analyzed the effect of shield excavation on foundation settlement by applying a vertical permanent load on the top of the foundation, taking into account the self-weight of the building and the self-weight of the floor slab. Ning^7^ established a two-dimensional finite element model to study the impact of shield construction on buildings from both deformation and force aspects. He^8^ established a two-dimensional finite element model, used equivalent loads instead of buildings to act on the model, and derived the influence law of double-hole excavation on the ground surface and foundation settlement when close to the buildings. Imamura^9^ conducted centrifugal model tests of shield excavation support with a miniature shield machine, analysed the results of the experiments and then simulated the excavation process with finite element software, analysed the distribution of the surrounding earth pressure in the tunnels at different depths of burial conditions, and studied the settlement of the ground at the gap between the shield tail. Xu^10^ conducted the indoor model test to get the guidance to optimise the shield construction method, this process carried out the research on the adaptability between the main shield construction parameters such as jack thrust, cutter torque and the characteristics of the soil layer, as well as the matching relationship between the various shield construction parameters. Fan^11^ conducted an indoor excavation test in order to obtain the influence law of shield excavation on the surface settlement and the stability of sand and gravel, a miniature shield machine was used in the test simulation, and the stability of the excavation surface and the surface settlement law were investigated at different burial depths. Jun^12^ conducted indoor boring experiments to study the dynamic change law of tunnel construction on the soil, and quantitatively analyzed the effect of shield construction on the disturbance of other strata when over-excavation of soft soil is adopted.

With the development of computer technology, many researchers at home and abroad have gradually begun to use numerical simulation methods to study the stability of the shield tunnel excavation surface. Hansen and Clough^13^ analysed the effect of anisotropic soil layer on the surface settlement value and foundation wall displacement by numerical simulation, and the results of the study showed that: the anisotropic soil layer increases the surface settlement displacement and foundation wall displacement, and also increases the damage area. Jenck^14^ used Flac3D to simulate shield construction and buildings to study the effect of soil loss and building stiffness on surface displacement, and the study found that the surface settlement changed near the shield tunnel excavation through the buildings, and the corresponding areas need to be protected. Michael^15^ simulated the shield excavation process to compare three lining models: continuous lining without joints, lining with aligned joints, and lining with staggered (rotating) joints. Li^16^ carried out simulation of two construction methods, the upper and lower step method and CRD method, using ANSYS software to study the lateral and longitudinal settlement of the ground surface of the two construction methods. Li^17^ established a three-dimensional model of the tunnel underpass frame structure, studied the change rule of surface settlement under the combined action of the tunnel construction and the building, as well as the impact of the construction on the building. Sun^18^ created a three-dimensional mechanical computation model of TBM tunnel boring continuously underneath several existing buildings using numerical computation, and comprehensively analysed the spatial property effect of deformation of a single building by single tunnel boring of TBM tunnel.

In this paper, for Beijing Metro Line 17, firstly, detailed investigation data and construction monitoring data are collected, and then theoretical analysis and numerical simulation methods are used to study the influence of shield tunneling in the ground layer on the deformation of the adjacent buildings, focusing on the response laws of stratigraphic parameters, engineering roles and other influencing factors on surface settlement, building tilting rate and twisting deformation, with emphasis on the sensitivity analysis of the degree of deformation influence, and then corresponding deformation control measures are proposed.

## Overview of the project

Beijing Metro Line 17 is located in the eastern part of Beijing, with a total length of 49.7 km, connecting four administrative districts of Tongzhou, Dongcheng, Chaoyang and Changping. Among them, the design mileage of the section from Ciqu Station to Ciqu North Station is K2 + 906.542–K4 + 106.725, with a total length of 1200.183 m. There are many buildings along the interval, and the main buildings on both sides are bungalows, 2-storey private buildings, and part of 6-storey residences that have been planned and realised. The construction has a great impact on them. The distance between the left and right lines of the shield tunnel is 13–17.2 m, and the depth of the tunnel in the interval is about 7.91–17.2 m. The interval of K3 + 908.7–K4 + 9.5 is selected as the simulation section, with a total length of the tunnel of 100.8 m.

**Figure 1:**
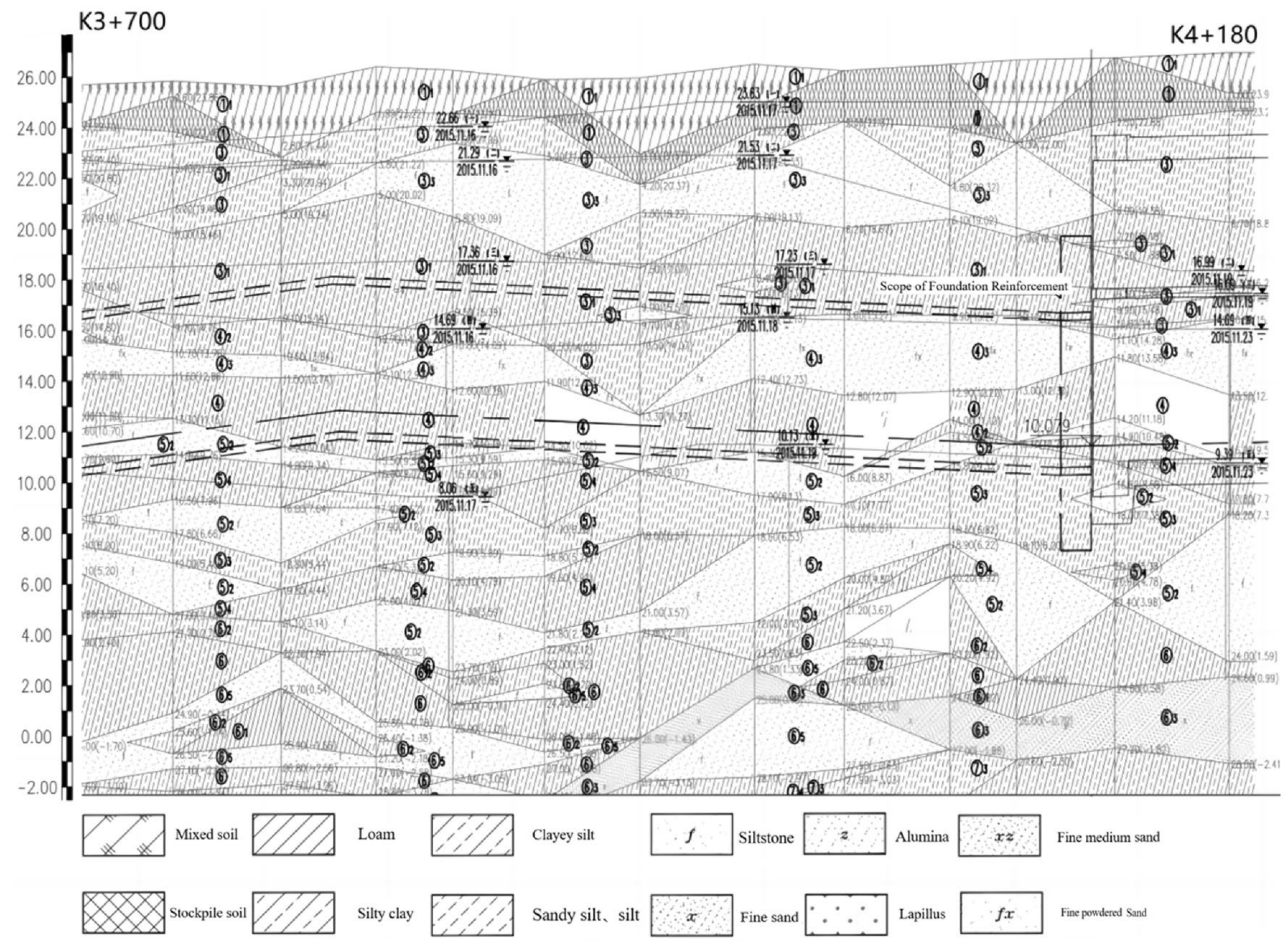
Tunnel profile view.

According to the drilling data and the results of indoor geotechnical experiments, the stratum is divided into 9 layers: ① plain fill, ①1 miscellaneous fill, ③ sandy silt, ③1 powdery clay, ③2 clay, ③3 silt, ④ powdery clay, ④2 clayey silt, ④3 powdery sand, ⑤2 powdery sand, ⑤3 sandy silt, ⑤4 powdery sand, ⑥ powdery clay, ⑥1 clay, ⑥2 clayey silt, ⑥3 fine sand, ⑥5 powdery sand, ⑦2 fine sand, ⑦3 sandy silt, ⑦4 powdery clay, ⑦5 clay, ⑧powdery clay, ⑧1 clay, ⑧2 clayey silt, ⑧3 powdery fine sand, ⑨1 fine medium sand, ⑨3 powdery clay. The longitudinal section of the tunnel is shown in Fig. 1.

Under the premise of implementing monitoring, according to the layer in front of the cutter plate, the shield thrust is controlled as much as possible after ensuring the soil pressure balance, which in turn controls the surface settlement in front of the shield. When the shield pushes forward, the soil pressure balance is established, the shield pushes forward smoothly and uniformly, the speed and the amount of soil output are strictly controlled, the pressure of the soil silo is adjusted, the pressure of synchronous grouting and the amount of grouting are controlled, and at the same time, the driving parameters are adjusted in time according to the changes of the strata to ensure that the loss of strata is minimized, so as to effectively control the elastic–plastic deformation of the strata. Strengthen the synchronous grouting and secondary grouting at the end of the shield to ensure that the void behind the pipe wall is filled, and to reduce the shrinkage of the rock surrounding the tunnel and the settlement of the ground layer. Shield tunneling parameters can refer to the following data: soil chamber pressure is 1–3 kg/cm^2^, thrust force is 1000–2000 t, cutter speed is 1–1.5 rpm, torque is 1500–3000 kN m, screw machine speed is 5–15 rpm, and soil boring speed is 40–60 mm/min.

## Numerical modelling

### Selection of model parameters

Within the modelled section, there is a 6-storey reinforced concrete office building 9.2 m from the right side of the tunnel, which poses a certain risk to the tunnel construction. In the simulation, the soil body is divided into four layers from top to bottom: plain fill, silt, powdery clay, clayey silt, and a homogeneous and equal-thickness isogenic layer is used to simulate the process of removing the shield shell, mixing the soil body and the cement slurry, and the thickness of the isogenic layer is taken to be 0.15 m. The values of the parameters of the soil layer, the tube sheet, the shield shell, and the isogenic layer are shown in Table 1.

**Table 1:** Model physical parameters.

Stratigraphic lithology (geology)	Thickness/m	Natural density/(kg m^−3^)	Cohesion/MPa	Angle of internal friction/^o^	Poisson's ratio	Modulus of elasticity/MPa
Stockpile soil	3.5	1800	0.018	16	0.3	13
Silt sand	2	1920	0.003	18	0.25	31
Silty clay	14.5	2100	0.021	20	0.3	26
Clayey silt	15	2100	0.031	27	0.3	26
Shield	0.15	7800			0.1	40,000
Tube sheet	0.3	2450			0.17	34,500
Isogenic layer	0.15	1850			0.2	100

### Numerical modelling

When establishing the model, the following basic assumptions are considered: (1) the material is homogeneous, continuous, and isotropic; (2) the effects of groundwater and seepage are not considered; (3) the initial stress only considers the gravity of the building and the soil; (4) during the simulation process, the friction and gravity effects of the shield machine on the soil are ignored; (5) the simulation ignores the time impact of construction and does not consider the creep of the soil; (6) the tunnel model is always parallel to the soil layer, regardless of the tunnel slope.

**Figure 2:**
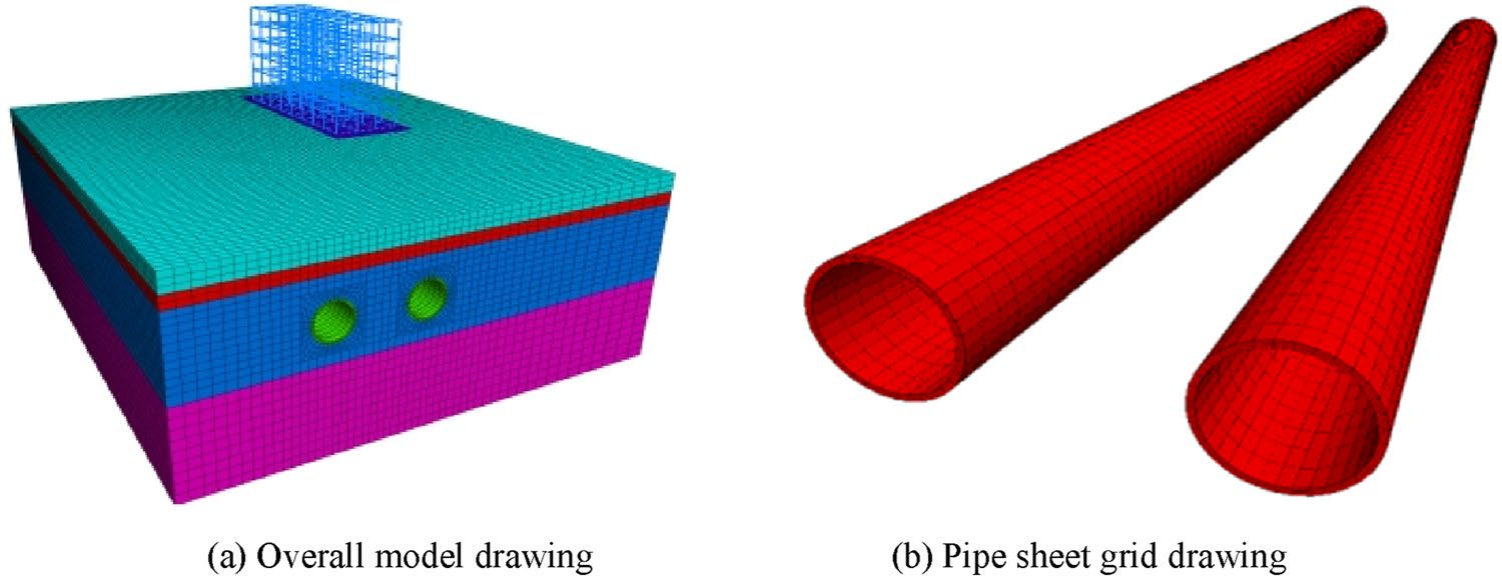
Numerical model.

During the simulation process, the entire soil layer was modeled using the Moulton-Coulomb model, the excavation face was modeled using an empty model, the building was modeled using a linear elastic model, and the segments and equivalent layers were modeled using an elastic model. The tunnel diameter was set at 6.1 m, the tunnel depth was 12.5 m, and the tunnel spacing was set to the actual spacing of 15 m. The right building’s pile foundation was an artificial excavation pile, with a distance of 9.2 m from the right tunnel. The pile bottom was 6 m vertically from the tunnel top. The model’s X-direction was 90 m long, and due to the presence of the right building, the horizontal direction from the right tunnel center to the right boundary was set at 47.5 m, while the horizontal direction from the left tunnel center to the left boundary was set at 27.5 m. The Y-direction was 100.8 m long, and the Z-direction was 35 m long. The numerical model contained a total of 160,860 elements and 166,600 nodes. The post-excavation model is shown in Fig. 2.

Prior to tunnel excavation, the soil layer is subject to self-weight stress. In order to truly reflect the real conditions of the site, it is necessary to simulate the initial stress field of the soil body before simulating the advance of the shield machine. The displacement caused by the stress is then reset to zero as the initial condition for studying the displacement and deformation of the layer during the advance of the shield machine. The formation of the initial stress field of the soil body can be achieved by setting the gravity acceleration condition for the model and performing an unbalanced iterative calculation to solve the initial stress field of the soil body. During the shield advance, the soil around the tunnel is disturbed, and the soil inside the tunnel is excavated and unloaded, which destroys the initial equilibrium state of the soil and leads to displacement and other problems. During the simulation process, iterative calculations are performed on the model, updating the unbalanced forces at each node. At each iteration, the unbalanced force at each node is multiplied by a release factor of 0.4 and then applied to the node in the opposite direction to release the stress.

### Comparative analysis of modelled and monitored surface settlement values

**Figure 3:**
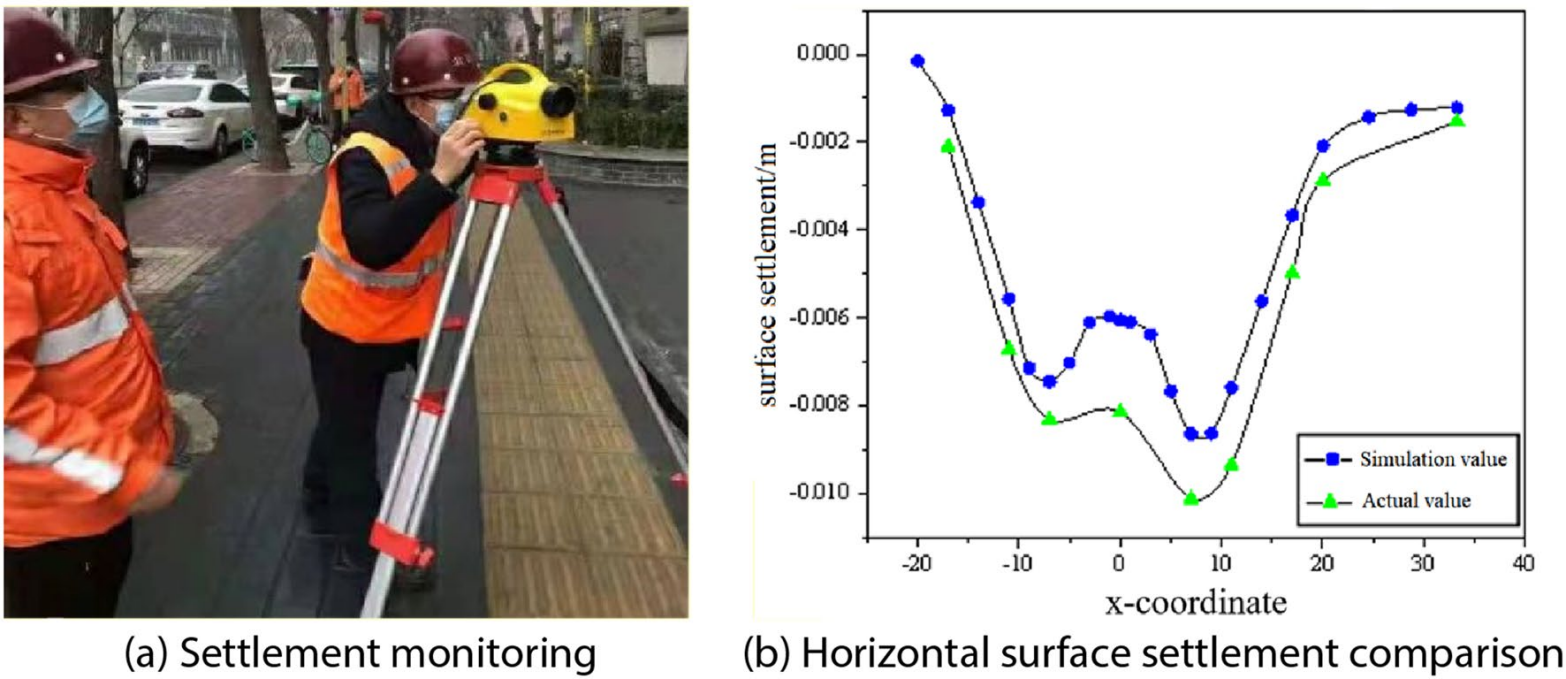
Monitor settlement and compare settlement value.

When arranging surface monitoring points, the spacing of measurement points is 10 m within 100 m of the starting well and 40 m of the arriving well, and 20 m for other ranges. Pipeline monitoring points are set up every 15 m, and surface monitoring points overlapping with pipeline monitoring points can be set up together. The main transverse monitoring section is arranged at the position of the liaison road, 10 m and 50 m from the starting well, and 10 m from the arriving well, with the spacing for other section ranges being 100–150 m. When encountering situations where monitoring cannot be set up, it can be fine-tuned locally according to the actual situation, and the monitoring results will be promptly fed back to the relevant units. Figure 3a shows the settlement monitoring sites.

The cross-sectional surface settlement from the shield excavation to 48 m was selected as the research object to derive the law of lateral surface settlement, and the actual monitoring values of lateral surface settlement were compared with the numerical simulation values, and the results are shown in Fig. 3b.

From the trend of the curve in Fig. 3b, it can be seen that the simulated lateral surface settlement and the actual lateral surface settlement curves have similar trends, the curves of the simulated value and the actual monitoring value are W-shaped, and the maximum settlement point occurs at the centre line of the right tunnel, with the simulated maximum value of 8.63 mm and the actual monitoring maximum value of 10.12 mm, with a difference of 1.49 mm, and the difference rate is 14.7%. Therefore, the simulation can reflect the actual shield construction process, and the subsequent simulation results are reliable and have reference value.

## Study on the deformation law of adjacent buildings caused by the dynamic boring of shield tunnel

### Mechanism of shielding effect on building deformation

**Figure 4:**
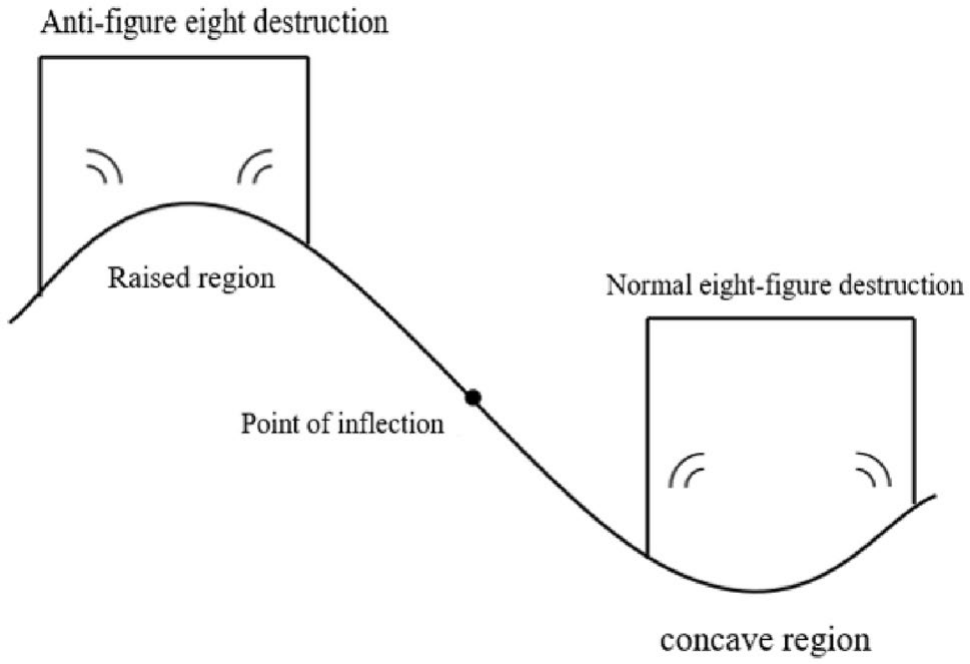
The effect of surface curvature on building deformation.

The reasons for the effect of shield tunnel excavation support on the deformation of surface structures can be divided into three areas:

1. Surface deformation: Shield tunnelling will have an impact on the ground, deforming the soil and causing the surface to bulge or settle, which in turn will create a curved surface, which is the surface curvature, as shown in Fig. 4. The occurrence of surface curvature means that the support of the surface on the building has changed.
2. Uneven settlement of the ground surface: in underground construction, uneven settlement of the ground surface must pay special attention to, if the tunnel depth is shallow, the soil layer is poor, it will produce a large uneven settlement, resulting in deformation of the foundation of the building damage, which in turn triggered the superstructure cracks, tilting, collapse and other damage.
3. Horizontal deformation of the ground surface: horizontal deformation of the soil body will have an effect on the lower foundation of the building, if the deformation is greater, it will produce a greater force on the foundation of the building, resulting in a greater deformation of the foundation, which will lead to damage to the building.

In summary, the deformation of the building is related to the curvature of the ground surface, the uneven settlement of the ground surface and the deformation of the horizontal direction of the ground surface. During the process of tunnel excavation and support, regardless of the ground conditions, building rigidity, and tunnel support measures, tunnel construction will damage the soil stress distribution to varying degrees, resulting in deformation and even damage to adjacent buildings. The impact of tunnel construction on buildings is a dynamic process. When construction reaches the nearest to the buildings, it is often the most unfavorable state for the buildings. Therefore, comprehensive consideration is needed to study the deformation impact of shield tunneling on adjacent buildings. In the construction process, it should be prevented and controlled as much as possible to avoid damage to the building due to excessive deformation and to reduce the damage caused by tunnel excavation and support to neighbouring buildings.

### Investigation of the surface settlement law in shield tunnelling

**Figure 5:**
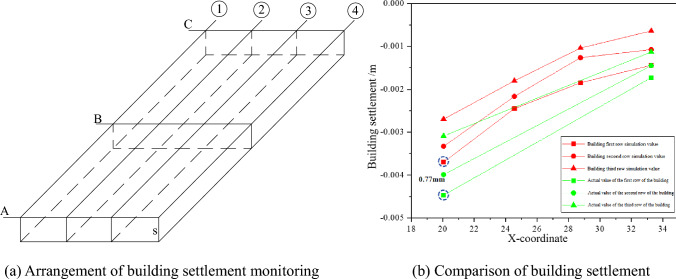
Arrange monitoring points and compare settlement.

In the actual engineering monitoring, three rows of settlement monitoring points are set for the building, as shown in Fig. 5a, where A is the first row of monitoring section for the building, B is the second row of monitoring section for the building, C is the third row of monitoring section for the building, and S is the value of settlement of the building. The settlement comparison of the three rows of monitoring points is shown in Fig. 5b.

From Fig. 5b, it can be seen that the simulated settlement of the building and the actual monitoring settlement of the building have similar trends. The maximum difference occurred within the first monitoring section at 0.77 mm. Through the analysis of the above figure, the simulated settlement of the building is consistent with the actual monitored settlement of the building.

**Figure 6:**
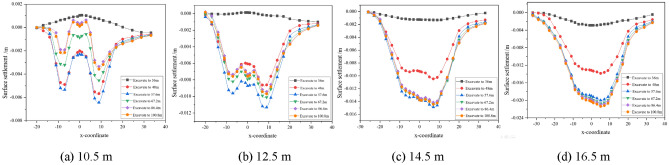
Dynamic settlement of monitoring points at different burial depths.

**Figure 7:**
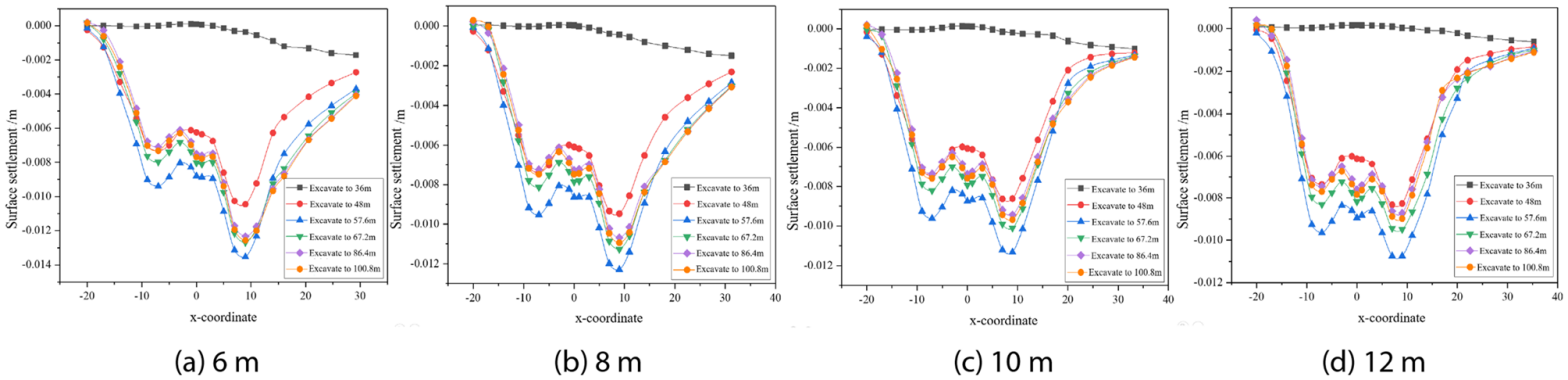
Dynamic settlement of monitoring points at different intervals.

**Figure 8:**
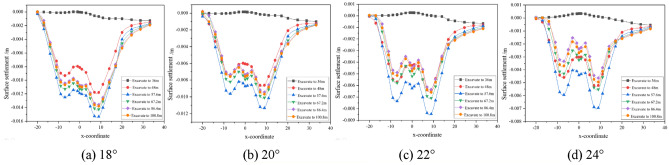
Dynamic settlement of monitoring points at different internal friction angles.

The effects of burial depth, spacing and internal friction angle on the settlement of the surrounding surface are discussed separately. To be more in line with the actual situation in the project, the simulation process takes into account the burial depth, spacing and internal friction angle to simulate four types of tunnel burial conditions, as shown in Figs. 6, 7, 8, and Table 2 shows the maximum surface settlement values corresponding to each condition.

**Table 2:** Maximum settlement under different working conditions.

Considerations	Parameter value	Maximum building settlement/mm
Depth of burial/m	10.5	6.6
	12.5	11.3
	14.5	14.9
	16.5	21.6
Spacing/m	6	13.7
	8	12.3
	10	11.3
	12	10.9
Angle of internal friction/°	18	15.5
	20	11.3
	22	8.4
	24	6.8

As can be seen from Fig. 6, the increase in excavation depth from 14.5 to 16.5 m has the largest increase in surface settlement, i.e. the increase in excavation depth has an increasing influence on surface settlement. Boring to 36 m on its influence is small, it can be seen that tunnel excavation has a certain range of influence on its driving direction; surface settlement with the increase in depth of burial was first increased and then decreased, the depth of burial is relatively shallow, tunnel construction overburden pressure is smaller than the supporting force, the ground surface will rebound phenomenon in the process of construction; depth of burial of 14.5 m, 16.5 m, when the tunnel is bored into the 48 m, the value of settlement of the building incremental increase in large, the later boring the change of building settlement value is small.

As the distance decreases, the surface settlement gradually increases, and the increase also gradually increases, i.e. the influence of decreasing distance on the surface settlement increases. When the distance between the tunnel and the building is 12 m, the surface settlement of the left line is similar to that of the right line, and when the distance is greater, the building has less influence on the tunnel, which is relatively safe, and the support can be reduced accordingly to save cost under such conditions. Therefore, after tunnel boring to 57.6 m, the surface settlement rebound is small, and the smaller the distance, the smaller the rebound, after tunnel boring to 67.2 m, the relative settlement value of the building does not change much.

From Fig. 8, it can be seen that as the internal friction angle decreases, the surface settlement gradually increases and so does the increase, i.e. the influence of decreasing the internal friction angle on the surface settlement is increased. With the increase of the angle of internal friction, the change of the value of the building settlement is small after the tunnelling to 67.2 m.

### Effect of dynamic shield tunnelling on the slope of the structure

According to the relevant standards^19^ can be understood: for the multi-storey frame structure of the building deformation by the settlement difference of adjacent columns analysis and calculation, the overall tilt rate of the building is the tendency of the building between the two points of the foundation settlement difference and the ratio of the distance between the two points. The local tilt rate of the building is the ratio of the settlement difference between two points and the distance between these two points in the building foundation selected between 6 and 10 m. The total tilt rate of the building in the longitudinal and transverse directions is taken as the object of study.

#### Effect of different working conditions on the longitudinal tilt rate of buildings

**Figure 9:**
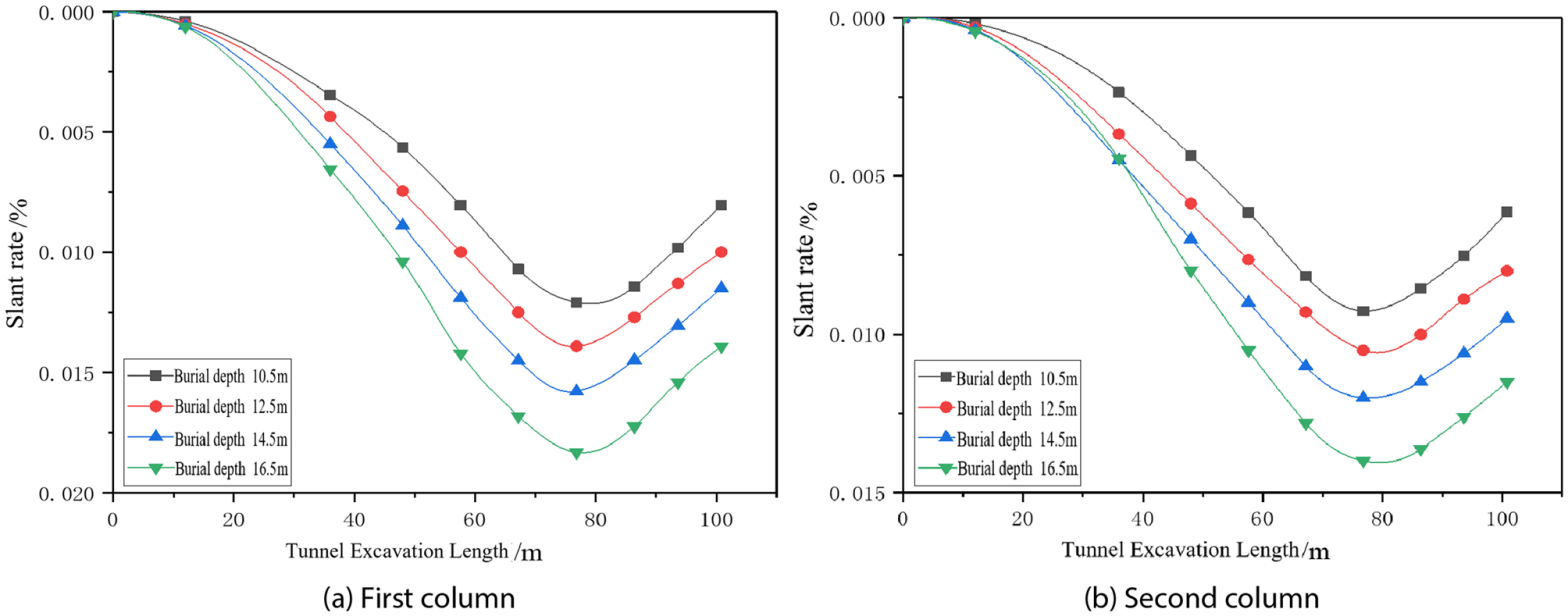
Dynamic longitudinal tilt rate of buildings at different buried depth.

**Figure 10:**
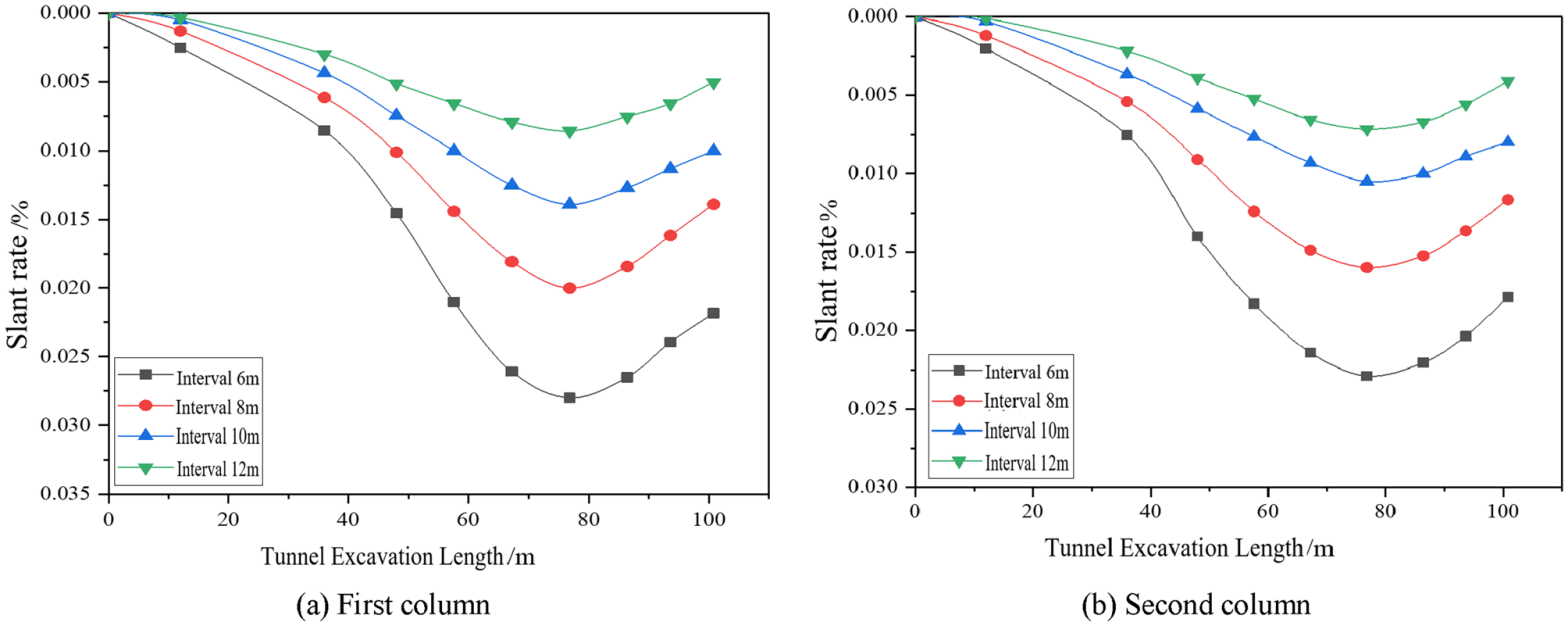
Dynamic longitudinal tilt rate of buildings at different distances.

**Figure 11:**
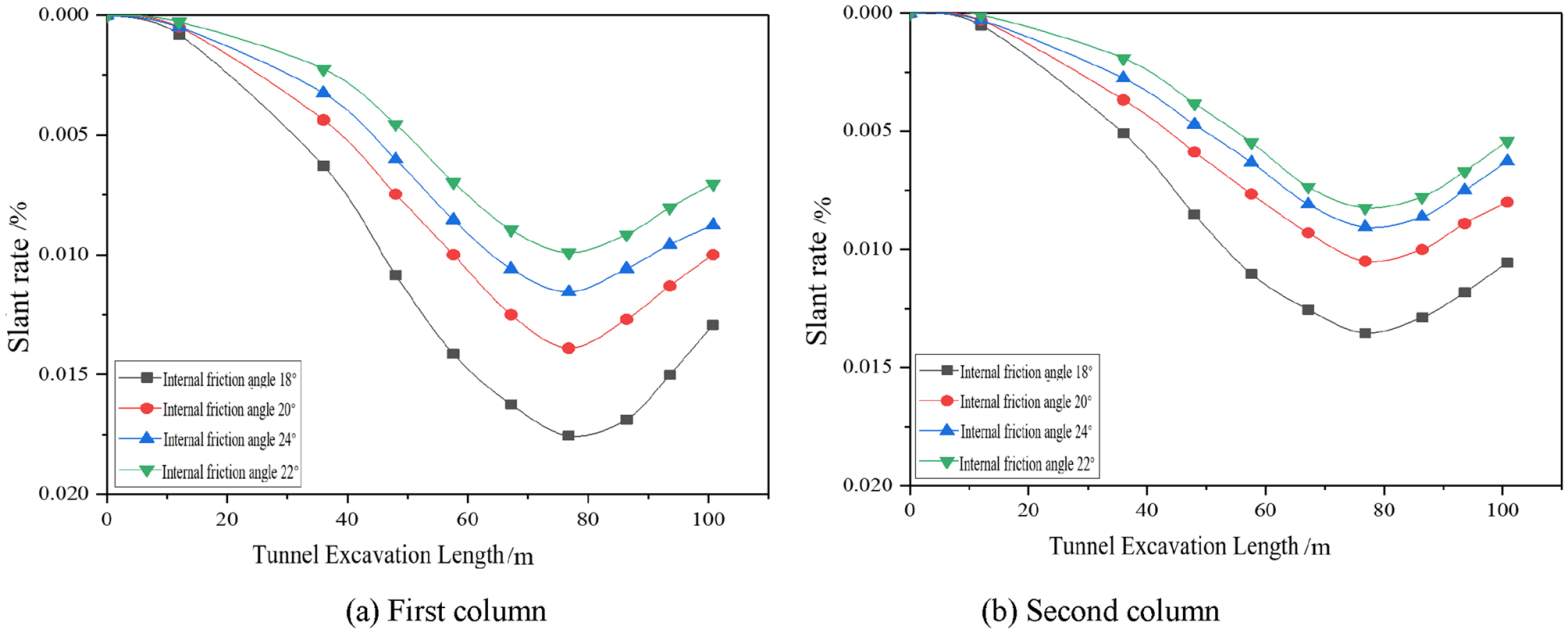
The dynamic longitudinal tilt rate of the building under different internal friction angles.

The settlement of the pile bottom was used to calculate the tilt rate of the building, and the effects of various factors on the longitudinal tilt rate of the building were analysed by the settlement deformation of the pile bottom corresponding to the settlement monitoring points in the first and fourth columns (the second column of the actual measurement) in the arrangement of the settlement monitoring points of the building shown in Fig. 5a, as shown in Figs. 9, 10, 11.

The longitudinal inclination rate of the two columns of the building has the same trend of change, which increases and then decreases with tunnel boring; the shield construction really affects the settlement and deformation of the building when boring to 12 m, and the inclination rate of the building reaches the maximum when boring to 76.8 m, and the symmetry axis can be obtained from the development trend of the building to be 76.8 m. The longitudinal tilt rate increases with the increase in burial depth, and increases with the decrease in spacing and internal friction angle; the change in spacing has the greatest influence on the longitudinal tilt rate of the buildings.

#### Effect of different working conditions on the lateral tilt rate of the building

**Figure 12:**
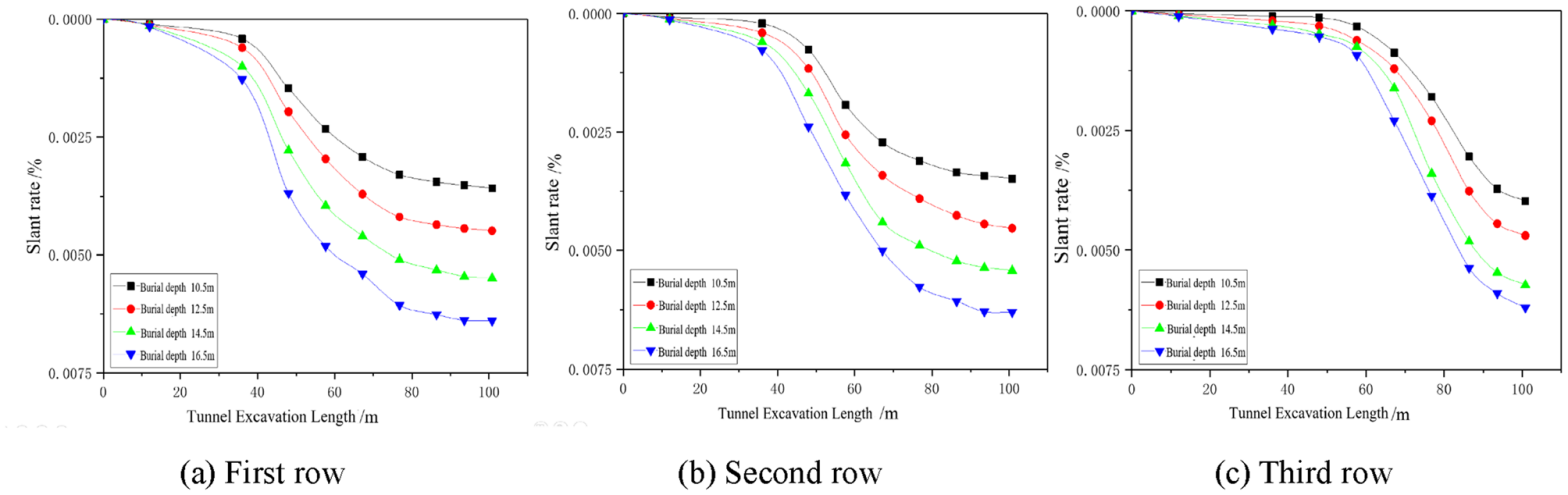
Dynamic lateral tilt rate of buildings at different buried depth.

**Figure 13:**
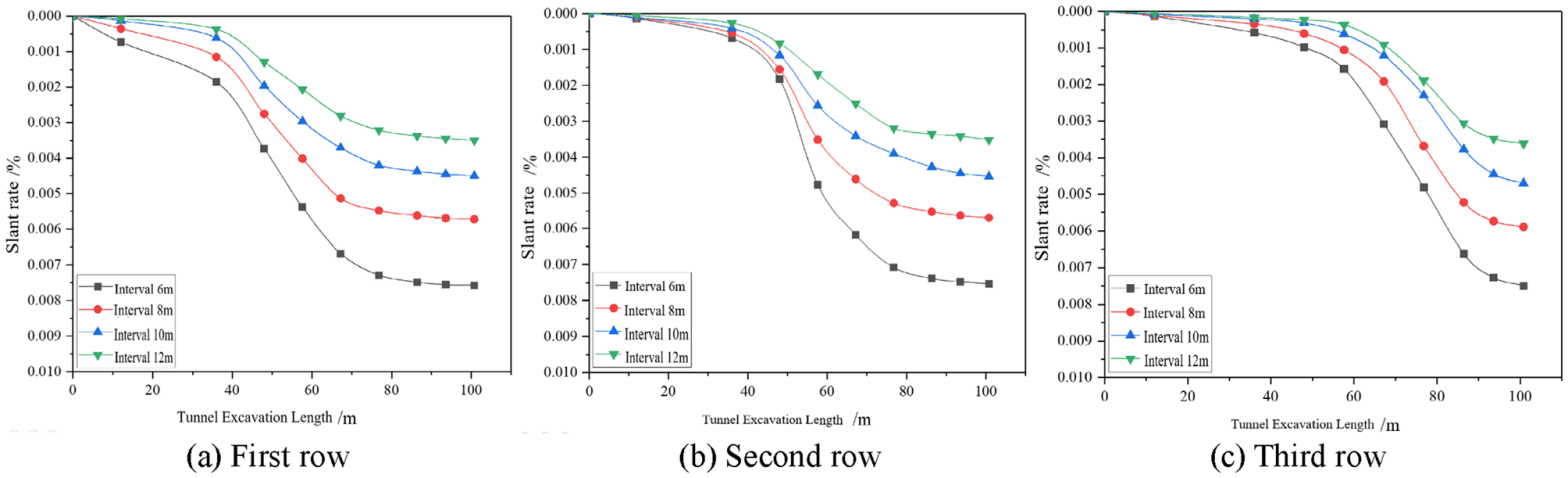
Dynamic lateral tilt rate of buildings at different distances.

**Figure 14:**
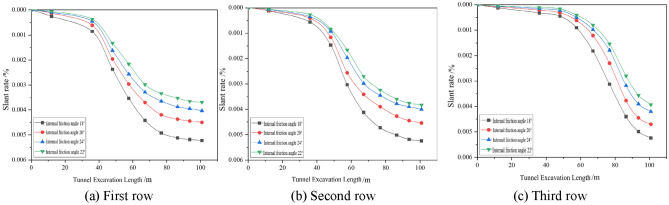
Dynamic lateral tilt rate of buildings under different internal friction angles.

The effects of different factors on the lateral inclination rate of the building are analysed by the settlement deformation of the pile bottom corresponding to the three rows of settlement monitoring points in the building settlement monitoring point arrangement diagram shown in Fig. 5a, as shown in Figs. 12, 13, 14. Due to the different locations of the monitoring sections, the lateral inclination rate of each section starts to change at different excavation faces, and the lateral inclination rate increases significantly after the first and third sections are driven to 36 m, 48 m and 67.2 m, respectively, but the final values of the lateral inclination rate of the three rows are basically the same under the same working conditions. Under different working conditions, the lateral inclination rates of the monitoring points in the three rows of sections increase gradually with the advance of the tunnel. The lateral inclination rate increases with increasing excavation depth and increases with decreasing spacing and internal friction angle.

### Impact of dynamic shield tunneling on twisting deformation of buildings

The displacement and deformation data obtained from the numerical simulation of the building with the tunnel boring, combined with the building twisting deformation formula^20^, can be obtained with the tunnel boring of the building twisting deformation of the rule of change.

1 $${T}_{w}=\frac{\left({S}_{A1}-{S}_{C1}\right)-\left({S}_{A4}-{S}_{C4}\right)}{B\cdot L}$$

where: *T*_*w*_ is the twisting deformation of the building; *S*_*A1*_, *S*_*C1*_, *S*_*A4*_, *S*_*C4*_ are the settlement values of the building at the four corner points; B is the distance from section A to section C; *L* is the distance from one column to four columns.

**Figure 15:**
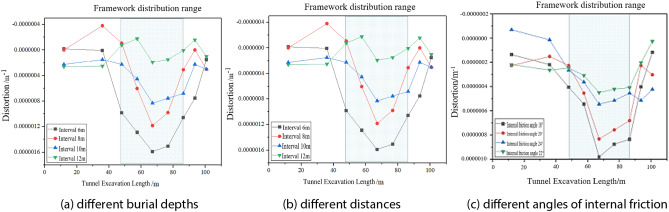
Dynamic distortion and deformation of buildings under different working conditions.

As can be seen in Fig. 15, the twisted deformation of the building is basically zero; when the shield tunneling to 48–86.4 m interval (shaded area) when the twisted deformation of the building is relatively large; when the shield tunneling to 67.2 m, the twisted deformation of the building reaches the peak (settlement and settlement difference of the building is not the maximum), the depth of burial of 10.5 m (the smallest depth), the twisted deformation of the building is not much change; visible shield tunneling on the twisted deformation of the building is not the deeper the depth of burial on its influence, but mainly depends on the settlement difference of the building; buildings and tunnels the greater the distance between the building will be gradually away from the tunnel excavation induced by the ground produced by the settlement slot, so that the building twisted deformation caused by the excavation of the influence of the smaller, and the maximum twisted deformation is also the smaller, in the area of the settlement slot, must pay attention to the building twisted deformation of the damaging effects, and reinforcement and protection measures must be taken; the three working conditions have little change on the influence of building twisted deformation.

### Sensitivity analysis of shield tunnel impact factors on adjacent buildings

The maximum settlement of the building is taken as the object of study to analyse the degree of influence of each of the shield tunneling factors on the existing building. The sensitivity of the factors must be determined by the sensitivity coefficient, as shown in Eq. (2).

2 $$ \left\{ {\begin{array}{ll} {S = {{\eta_{1} }/{\eta_{2} }}} \\ {\eta_{1} = {{\left| {\Delta F_{s} } \right|} /{F_{so} }}} \\ {\eta_{2} = {{\left| {\Delta X} \right|} /{\left( {X_{\max } - X_{\min } } \right)}}} \\ \end{array} } \right. $$

where:$\left| {\Delta F_{s} } \right|$ is the change of the maximum settlement value of the building caused by the change of a single factor;$F_{so}$ is the maximum settlement value of the building solved for the basic working condition;$\left| {\Delta X} \right|$ is the change of the factor;$X_{\max } - X_{\min }$ is the difference between the maximum value and the minimum value of a single factor.

**Figure 16:**
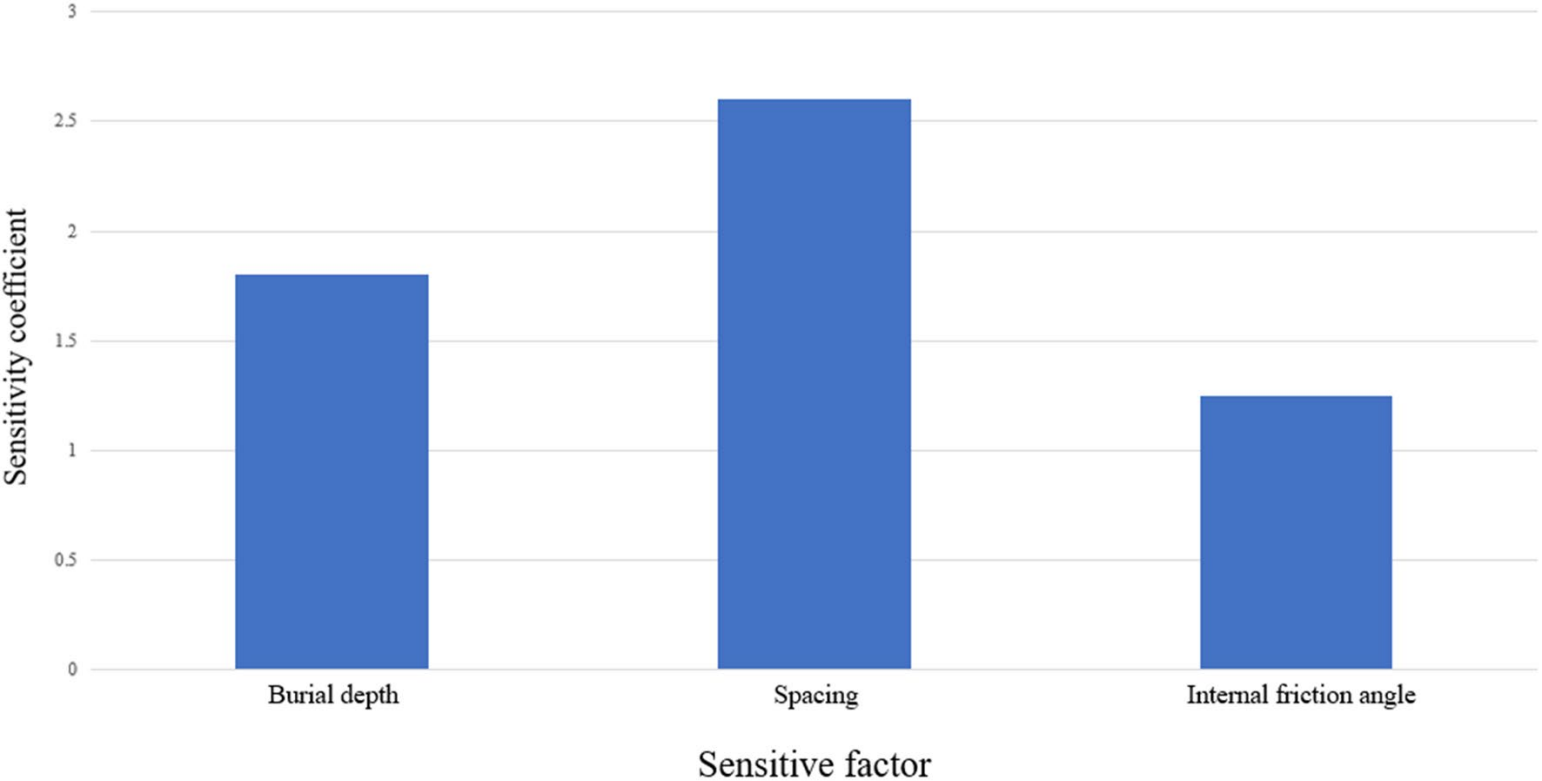
Histogram of sensitivity coefficient.

The calculation results of the maximum settlement change rate of each influencing factor are shown in Table 3, and the calculation results of the sensitivity coefficient of each influencing factor are shown in Fig. 16. The order of sensitivity of the factors influencing the deformation of the building is: spacing > depth of burial > angle of internal friction.

**Table 3:** Maximum settlement change rate of buildings.

Considerations	Parameter value	Maximum building settlement/mm	Rate of change/%
Depth of burial/m	10.5	1.41	61.89
	12.5	3.7	0
	14.5	4.91	32.7
	16.5	7.14	92.97
Spacing/m	6	8.8	137.84
	8	6.86	85.41
	10	3.7	0
	12	2.08	43.78
Angle of internal friction (°)	18	5.28	42.7
	20	3.7	0
	22	2.11	42.97
	24	1.59	57.03

## Conclusion

Clay layer shield tunnel adjacent to the building deformation is closely related to the depth of burial, hole diameter and other factors that affect the deformation of the building sensitivity ranking: spacing > depth of burial > angle of internal friction, depth of burial and spacing of the building deformation is a strong sensitivity factors, in the large depth of burial, small angle of friction, the building is close to the tunnel, must pay special attention to the protection of the deformation of the building.

1. When the tunnel is excavated to 36 m, the impact on the surface settlement of monitoring section I is relatively small; when the burial depth is shallow, the surface will rebound during the tunnel excavation process; the dynamic excavation of deep-buried tunnels will result in a large increase in the settlement value of buildings in the early stage, and the settlement value of buildings will change slightly in the later stage.
2. The longitudinal inclination rates of the two rows of buildings have the same trend of change, both increasing and then decreasing with the tunnel excavation; when the shield is excavated to 76.8 m, the longitudinal inclination rate of the building reaches its maximum value, and from its development trend, its symmetry axis is 76.8 m; the change in the distance between the tunnel and the building has the greatest impact on the longitudinal inclination rate of the building. Under the same working conditions, the final values of the three rows of lateral inclination rates are basically the same; under different working conditions, the three rows of lateral inclination rates gradually increase with the tunnel excavation.
3. The distortion and deformation of buildings caused by shield tunneling mainly depends on the differential settlement of the buildings. When the distance between the buildings and the tunnel is small, it is necessary to pay attention to the destructive effects caused by the distortion and deformation of the buildings and take reinforcement and protection measures. When the distance between the tunnel and the buildings is large, the change in internal friction angle has a small impact on the distortion and deformation of the buildings.

## Data Availability

The datasets generated during and analyzed during the current study areavailable from the corresponding author (Feng Jiang) on reasonable request.
